# Biological maturation drives the hepatic-to-renal switch in erythropoietin production at birth

**DOI:** 10.1016/j.ebiom.2026.106277

**Published:** 2026-05-05

**Authors:** Salam Idriss, Laurent Martin, Nada Maaziz, Esther Juguet, Denis Semama, Laure Merle, Stuart S. Winter, Amandine Caillaud, Alexandre Marchand, François Girodon, Betty Gardie

**Affiliations:** a Nantes Université, CNRS, INSERM, L’institut du Thorax, Nantes F-44000, France; b Laboratoire Antidopage Français (LADF), Université Paris-Saclay, Orsay, France; c Service d’Hématologie Biologique, Pôle Biologie, CHU de Dijon, Dijon, France; d Réanimation Néonatale, Service de Pédiatrie, CHU Dijon, Dijon, France; e Department of Pediatrics, University of New Mexico Comprehensive Cancer Center, Albuquerque, NM, USA; f Laboratoire d’Excellence GR-Ex, Paris, France; g EPHE, PSL Université, Paris, France

**Keywords:** Erythropoietin, Neonates, Hepatic-to-renal switch, Liver organoids, Hypoxia

## Abstract

**Background:**

Erythropoietin (EPO) is essential for erythropoiesis, with production shifting from the foetal liver to the kidney after birth. This hepatic-to-renal transition is a hallmark of neonatal adaptation, but the relative contribution of oxygen exposure *versus* intrinsic developmental processes remains unclear.

**Methods:**

We studied plasma EPO isoform profiles in 89 neonates (developmental age 29–56 weeks; chronological age 1–92 days). Isoforms were classified as hepatic-like or renal-like based on isoelectric focusing patterns. Profiles were correlated with developmental and chronological age to assess the influence of oxygen exposure *versus* maturation. In parallel, human induced pluripotent stem cells (hiPSCs) were differentiated into liver organoids and cultured under hypoxic or normoxic conditions. Transcriptome and *EPO* mRNA expression were measured throughout differentiation to evaluate developmental and oxygen-dependent regulation.

**Findings:**

Neonatal EPO profiles shifted progressively from hepatic-like to renal-like with increasing developmental age. This transition was independent of time spent in atmospheric oxygen; preterm infants retained hepatic-like profiles for weeks after birth despite continuous oxygen exposure. *In cellulo*, immature hepatocytes expressed *EPO* robustly under hypoxia, but expression declined sharply as hepatocyte maturation advanced and was absent at later stages, regardless of oxygen tension. Maintaining the immature state of hepatic cells may be critical for responsiveness to hypoxia and for EPO production.

**Interpretation:**

These findings indicate that intrinsic hepatic maturation, rather than oxygenation, governs the silencing of hepatic-derived EPO after birth. Clarifying this process could support novel therapeutic strategies aimed at reactivating foetal-like hepatic EPO production to treat anaemia, particularly in preterm infants and in conditions associated with insufficient renal EPO production, such as chronic kidney disease.

**Funding:**

This study was mainly supported by the Région des Pays de la Loire, The 10.13039/501100001665French National Agency for Research (ANR), the Fonds Européen de Développement Régional Bourgogne Franche Comté, the Marie Skłodowska-Curie action, and the Fondation Génavie.

**Research in context**

**Evidence before this study**

In the literature, animal studies have shown that the foetal liver is the major site of EPO production before birth, whereas the kidney becomes predominant postnatally. In humans, indirect evidence of foetal hepatic EPO production has been reported in neonates with renal agenesis, who nevertheless had high circulating EPO levels. Prior work suggested that the postnatal rise in oxygenation contributes to the physiological decline in hepatic EPO production, but it remained unclear whether oxygen exposure alone or intrinsic developmental cues govern the hepatic-to-renal switch. Evidence to date has been limited by reliance on indirect measures and by the lack of detailed analyses of EPO isoforms or of human developmental models. As a result, the developmental timing and regulatory mechanisms of hepatic EPO silencing in humans have remained poorly defined.

**Added value of this study**

This study provides direct evidence that the hepatic-to-renal switch in EPO production is driven primarily by intrinsic developmental maturation rather than postnatal oxygen exposure. By profiling EPO isoforms in 89 neonates of varying gestational and chronological ages, we show that the transition from hepatic-like to renal-like EPO profiles correlates with developmental age but not with duration of atmospheric oxygen exposure. Complementary experiments in hiPSC-derived liver organoids further demonstrated robust hypoxia-inducible *EPO* expression at early stages of hepatocyte differentiation, followed by a marked decline as hepatocytes matured, irrespective of oxygen tension. Together, these findings support a role for hepatic maturation as a key regulatory component contributing to postnatal hepatic EPO silencing.

**Implications of all the available evidence**

Our findings refine the current understanding of erythropoiesis during the neonatal period, demonstrating that developmental regulation rather than oxygen exposure drives the hepatic-to-renal switch in EPO production. This insight opens promising opportunities to explore transcriptional and epigenetic regulators of hepatic EPO silencing as potential therapeutic targets. Reactivating foetal-like hepatic *EPO* expression could represent a novel strategy to treat anaemia in preterm infants during critical developmental window and in patients with chronic kidney disease, potentially reducing dependence on exogenous EPO therapy and offering safer and more durable alternatives.

## Introduction

The discovery of the regulation of red blood cell production by oxygen levels, followed by the identification of the erythropoietin (*EPO*) gene in 1985,^1^ marked a major breakthrough in our understanding of the hypoxia pathway mechanisms, and the development of efficient anti-anaemic drugs. However, 40 years after the identification of the *EPO* gene, many aspects of its regulation remain undiscovered.^2^ The protein itself is heavily glycosylated with a diverse and complex glycans pattern. These glycosylations play a decisive role in biochemical properties and circulatory half-life of EPO protein.^3^ Yet, replicating the exact glycosylated forms found in human plasma *ex vivo* remains a significant challenge.^4^

EPO is a major regulator of erythropoiesis, and research on the regulation of *EPO* gene expression has a long history. Experiments in animals showed that the foetal liver is the primary site of EPO synthesis^5^^,^^6^ during development, whereas in adults, the kidney is the predominant source of EPO.^7^^,^^8^ In humans, circumstantial evidence for hepatic EPO production during foetal life comes from observations that foetuses or neonates suffering from bilateral renal agenesis had normal, even elevated EPO serum levels.^9^ The precise embryonic origin of EPO-producing cells, which may include migrating neural crest cells^10^^,^^11^ and the identity of renal interstitial EPO-producing cells in the kidney were only described decades later.^12^, ^13^, ^14^

The key regulatory elements that control *EPO* gene expression, in the kidney and liver, have been extensively studied. The Hypoxia Inducible Factor (HIF), through its binding to Hypoxia-Responsive Elements (HREs) in the 5′ and 3′ distal regions of the *EPO* gene, plays a central role in this regulation.^15^, ^16^, ^17^ In sheep, the switch in the site of EPO production from the liver to the kidneys is accompanied by a gradual decrease in hepatic EPO production,^18^ which is typically completed by the sixth week after birth. Beyond this point, the liver contributes only about 10–15% to total EPO production.^18^^,^^19^ It is considered that a similar process occurs in humans, nonetheless, little is known about the mechanisms underlying the developmental shift of EPO production from the foetal liver to the adult kidneys.

It is well established that the foetus develops under low oxygen tension, which is essential for proper embryonic development. This relative hypoxia ensures efficient oxygen unloading despite the high oxygen affinity of foetal haemoglobin (HbF) and plays a key role in promoting angiogenesis. Moreover, the foetal liver experiences even lower oxygen levels due to the placental circulation bypassing hepatic tissues *via* the ductus venosus.^20^ This particular hypoxic environment supports the liver’s role as the primary EPO-producing organ *in utero*. At birth, there is a significant increase in serum oxygen concentration due to a transition from placental to pulmonary respiration. This sudden shift in circulation and oxygenation coincides with a postnatal decline in plasma EPO level^18^^,^^21^ and a decrease in haemoglobin levels, typically observed in newborns within days to weeks after birth.^22^, ^23^, ^24^ This phenomenon could be related to the transitional shift from hepatic to renal EPO production. However, whether this shift is driven solely by increased oxygen availability or involves additional developmental factors/mechanisms remains unclear. Recent work in the developing kidney further supports the concept that EPO production can be developmentally constrained independently of systemic oxygen availability. In preterm neonates, tubular immaturity is associated with reduced oxygen consumption, higher local tissue oxygenation, and limited EPO production despite preserved hypoxia signalling capacity.^25^ These observations highlight the importance of developmental tissue physiology and metabolic state in shaping EPO regulatory competence.

Recently, our research group demonstrated that neonatal EPO isoforms can be distinguished from adult isoforms using isoelectric focusing (IEF-PAGE),^26^ an analytical technique that separates EPO proteins based on their charge and glycosylation patterns, consistent with previous studies.^27^^,^^28^ These differences are attributable to variations in the glycan content and strongly suggest a specific glycosylation pattern characteristic to liver-derived EPO. In addition, cord blood EPO, that can be considered “hepatic-like” as the blood comes mainly from the newborn, demonstrated a higher activity in stimulating the EPO receptor.^26^

In light of these discoveries, this study aimed to determine whether the postnatal transition of EPO production from the liver to the kidney is primarily associated with birth-related exposure to atmospheric oxygen or with intrinsic developmental maturation. To address this, we analysed EPO isoelectric profiles in neonates at different developmental stages and varying postnatal ages (i.e., time since birth and exposure to atmospheric oxygen), to evaluate whether these profiles reflected a hepatic, renal, or mixed origin. In parallel, using a human cellular model, we examined whether hepatic *EPO* expression changes during developmental maturation. We analysed *EPO* expression and transcriptomic profiles under normoxic and hypoxic conditions during the differentiation and maturation of human induced pluripotent stem cells (hiPSCs) into multicellular liver organoids.

## Methods

### Newborn’s age definitions

Age definitions used in this study are as follows: “Gestational age” is defined as the time elapsed between the first day of the last maternal menstrual period and the day of delivery, expressed in weeks of maternal amenorrhoea (WA). “Chronological age” refers to the time elapsed since birth, corresponding to extrauterine life and postnatal exposure to atmospheric oxygen, and is expressed in days, weeks, or months. “Developmental age” corresponds to the sum of gestational age and chronological age, and is expressed in weeks. It should be noted that gestational age includes approximately 14 days prior to conception, and therefore actual foetal development begins around two weeks after the calculated gestational age.

### Ethics, sample collection and analysis of neonatal plasma EPO isoforms

Residual plasma samples and associated data were collected retrospectively and processed in an anonymised manner. According to French regulations, this study qualifies as research not involving human subjects (RNIPH), as it is based exclusively on the reuse of existing clinical data and residual biological samples. In accordance with the methodology MR-004 of the CNIL, such studies do not require approval from an ethics committee. The study protocol (ClinicalTrials.gov identifier: NCT03957863) was reviewed by the institutional clinical research department of the University Hospital of Dijon. All procedures were conducted in compliance with the French Data Protection Act (Law No. 78-17 of January 6, 1978, as amended) and the General Data Protection Regulation (EU 2016/679). Parental consent was obtained where required.

Residual plasma samples from newborns (preterm < 37 weeks, and term 37< birth <42 weeks of developmental age as defined above) were collected shortly after birth up to a few months later for our correlations using chronological age. Plasma samples were stored frozen and subsequently sent for analysis at the French Anti-Doping Laboratory (University Paris-Saclay, France), which has extensive expertise in EPO glycoform characterisation by IEF-PAGE, a technique routinely used for the detection of EPO doping in athletes.

### Erythropoietin analysis

The profiles of EPO present in the patient plasma samples were assessed using the IEF-PAGE method, as previously described.^26^^,^^29^ Briefly, after specific immunopurification of EPO from plasma using magnetic beads coated with an anti-EPO antibody (clone 9C21D11, AB_2830015), the purified EPO isoforms were separated on a 2–6 pH gradient polyacrylamide gel. The proteins were transferred onto a PVDF membrane, which was then incubated with another specific anti-EPO antibody (clone AE7A5, AB_981030) to interrogate the EPO profile. In IEF-PAGE analysis, the human EPO profiles from healthy adults typically comprises several bands (11–16 bands, each representing several EPO isoforms with identical isoelectric point). These bands span from the acidic to the basic region, with the most intense bands normally observed in the “neutral region”. This region is defined by the exclusion of the acidic and basic regions, which correspond to the migrating positions of the isoforms from recombinant EPO drugs (darbepoetin alfa, specific to the acidic region, and epoetin alfa/beta, specific to the basic region). Band intensity quantification was performed using GASepo, a software tool dedicated to EPO image analysis for antidoping applications.^30^

### Statistics

Statistical analyses on IEF profiles were performed using GraphPad Prism (SCR_002798). Differences in profile distribution across age groups were assessed using a chi-square test of independence, with Fisher’s exact test used where appropriate. Age groups were treated as ordered, and changes in profile distribution across age were examined using proportional (percentage-based) plot.

### Differentiation of hiPSC into liver organoids

HiPSCs were generated from peripheral blood mononuclear cells (PBMCs) in the iPSC core facility of Nantes University. PBMCs were reprogrammed using Sendai viruses expressing OCT4, SOX2, KLF4, and c-MYC (CytoTune™-iPS 2.0 Sendai Reprogramming Kit, Thermo Fisher Scientific). As these cell lines were generated in-house and are not deposited in a public repository, no RRID is available. hiPSC clones were selected and expanded on mouse embryonic fibroblast (MEF) feeder cells in KSR-FGF2 medium (DMEM/F12 supplemented with 0.1% β-mercaptoethanol, 20% knockout serum replacement, 10 ng/mL basic fibroblast growth factor, 2 mmol/L L-glutamine, and 1% non-essential amino acids). Mycoplasma contamination was routinely excluded using the MycoAlert™ kit (Lonza, LT07-318). Reprogramming was validated by RT–qPCR.

Using a protocol adapted from Harrison et al.,^31^ liver organoids were generated from hiPSCs following a 21-day differentiation protocol, at 37 °C and 5% CO2. Up to 10 organoids were collected at various time points throughout the differentiation process to study temporal gene expression. Prior to each collection, organoids were cultured for 24 h in either normoxia or hypoxia (1% O_2_). Two independent series of differentiations were performed (n = 3 biological replicates per series), due to the limited number of organoids available at each time point. One series was dedicated to targeted gene expression analysis using TaqMan assays (details provided in Supplementary Data), and the second series was used for bulk 3′ RNA sequencing (3′ SRP). Total RNA was extracted from liver organoid differentiation samples using the NucleoSpin RNA kit (Macherey–Nagel). RNA quality and integrity were assessed using a NanoDrop spectrophotometer and the 4200 TapeStation System (Agilent Technologies).

### Quantification of gene expression by RT-qPCR during the differentiation of hiPSCs into liver organoids

RNA (1 μg) extracted from liver organoids was reverse transcribed into cDNA using the Maxima First Strand cDNA Synthesis Kit (Thermo Fisher Scientific). Quantitative polymerase chain reaction (RT-qPCR) was performed using Taqman® Universal PCR Master Mix (Applied Biosystems) on a QuantStudio 5 Real-Time PCR System (Applied Biosystems, SCR_020240). Hepatic differentiation was assessed by quantifying the expression of hepatic maturation markers using specific TaqMan probes: *ALB* (albumin, Hs00910225_m1), *CYP3A4* (Cytochrome P450 3A4, Hs00604506_m1) and *CYP3A7* (Cytochrome P450 3A7, Hs02511627_s1). The expression of *EPO* was measured using the TaqMan probe Hs00171267_m. Relative quantification of all genes was normalised to the mean expression of three reference genes, *RPLP0* (ribosomal protein lateral stalk subunit P0, Hs99999902_m1), *ACTB* (actin beta, Hs99999903_m1), and *RPL13A* (ribosomal protein L13a, Hs04194366_g1), using the 2^−ΔCt^ method.

### 3′-end sequencing RNA profiling (3′SRP) and data processing

3′ RNA-seq libraries preparation and sequencing were performed by the GenoA platform (Nantes Université) using the NovaSeq X Plus Sequencing System protocol.^32^^,^^33^ Libraries were prepared from 10 ng of total RNA using template-switching reverse transcription, allowing incorporation of sample-specific barcodes and unique molecular identifiers (UMIs) during poly(A) RNA capture. Barcoded cDNAs were pooled, amplified, and fragmented using a transposase-based tagmentation approach enriched for 3′ transcript ends.

For library preparation, 200 ng of full-length cDNA was processed using the Illumina DNA Prep Library Preparation kit (ref #20060060, Illumina) and Nextera XT Index Kit (ref #FC-131-1001, Illumina) according to the manufacturer’s instructions. Library size distribution was assessed using a 4200 TapeStation system (Agilent Technologies, SCR_018435). Libraries were sequenced on a NovaSeq X Plus platform using the NovaSeq X Series 1.5B Reagent Kit, 100 cycles (ref #20104703, Illumina) with a 19–8–103 cycle configuration (SCR_024568).

Raw sequencing data were processed using a Snakemake-based pipeline (3′ SRP). Reads were demultiplexed, aligned to the RefSeq transcriptome using BWA (SCR_010910), and quantified using UMI-based counting to generate gene-level expression matrices. Differential expression analysis was performed using DESeq2^34^ (SCR_015687), and functional enrichment analysis was conducted using ClusterProfiler (GO, KEGG, and GSEA) (SCR_016884). Quality control metrics, including sequencing depth and mapping rate, were assessed prior to downstream analysis.

### Transcriptomic analysis

Raw count data were processed using DESeq2 in R, applying two normalisation strategies: size factor normalisation (normalised counts) for individual gene expression visualisation and quantitative comparisons, and variance stabilising transformation (VST) for unsupervised exploratory analyses including principal component analysis (PCA) and marker-based cell population profiling. Cell type-specific temporal profiles were reconstructed using curated marker gene sets from the literature, covering major hepatic cell populations (hepatocytes, immature hepatocytes, cholangiocytes, Kupffer cells, liver sinusoidal endothelial cells, hepatic stellate cells), as well as hypoxia-responsive genes and transcription factors. At day 19, pairwise differential expression analysis between hypoxia and normoxia was performed using a full interaction model to identify significantly regulated genes based on adjusted p-values and log_2_ fold changes. Full details of the bioinformatic pipeline are provided in Supplementary Data.

### Role of funders

The funders had no role in the study design; data collection, analysis, or interpretation; manuscript preparation; or the decision to submit the manuscript for publication.

## Results

### Analysis of EPO IEF-PAGE profile in neonates across developmental ages and durations of postnatal exposure to atmospheric oxygen

Over a period of three years, 89 residual blood samples were collected from neonates, either born prematurely (of developmental age between 29 weeks and 36 weeks) or at full term ≥37 weeks. Following birth, a total of 34 plasma samples were obtained from neonates with developmental age ≤ 37 weeks, 27 samples from those between 37 and 41 weeks of developmental age, 16 samples from those between 41 and 49 weeks and 12 samples from neonates with developmental age over 49 weeks (Supplementary Table S1).

**Fig. 1:**
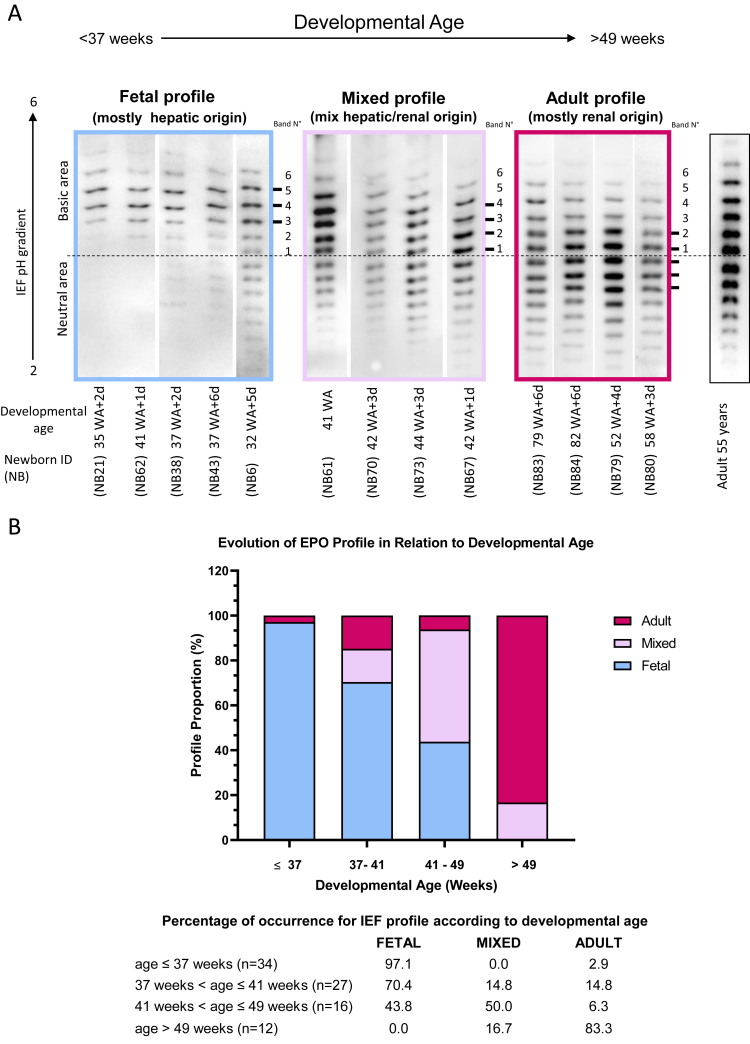
**Analysis of EPO isoforms in newborns at different developmental ages.** (A) Isoelectric focusing electrophoresis showing EPO isoform profiles in individual patient serum samples collected from newborns at varying developmental ages. Each lane represents a distinct patient sample. EPO profiles were classified based on the relative intensity and position of the predominant isoform bands. The foetal profile (EPO predominantly of hepatic origin) is characterised by the three most intense bands located at positions 3, 4, and 5. The mixed profile (mix of EPO from hepatic and renal origin) is characterised by the three most intense bands located at positions 2, 3, and 4 or positions 1, 2, and 3. The adult profile (EPO predominantly of renal origin) is characterised by the presence of one or more of the three most intense bands in the neutral region of the IEF gel, similar to the pattern observed in healthy adults. (B) Graphical representation of the changes in EPO isoform profiles as a function of developmental age (weeks). EPO profiles were categorised as foetal, mixed, or adult based on the most intense bands quantified in the EPO isoforms patterns observed in panel A. The distribution of foetal, mixed, and adult EPO profiles differed significantly between developmental age groups (chi-square test of independence, p < 0.0001). Abbreviations: WA, weeks of amenorrhoea; d, days; NB, newborn; EPO, erythropoietin; IEF-PAGE, isoelectric focusing–polyacrylamide gel electrophoresis.

To determine whether the transition of EPO expression from the liver to the kidney after birth is driven by developmental stage or by the duration of exposure to atmospheric oxygen, we analysed and compared EPO profiles from preterm and term neonates, either in the first week of life or after several weeks to months of breathing atmospheric oxygen. EPO profiles, obtained after separation by IEF-PAGE, showed variations in the position/distribution of the most intense EPO isoforms from a predominance in the basic area (similar to Hepatic-like EPO, as previously described^26^) to a majority of the signal in the neutral area (similar to healthy adult renal EPO profile). We then categorised the different migration profiles obtained from the 89 samples by numbering each band in the basic area of the gel from 1 to 6 and identifying the position of the three most intense bands. As shown in Fig. 1A, the profiles showing the most intense bands 3, 4 and 5 were classified as “foetal profiles”, consistent with predominant hepatic EPO production, as they majorily corresponded to preterm neonates. In contrast, profiles with one or more of the three most intense bands located in the neutral area were highly similar to healthy adult renal EPO profiles^26^ and were classified as “adult profile”, consistent with predominant renal EPO production. “Mixed profiles”, defined by the three most intense bands located at positions 2, 3, and 4 or positions 1, 2, and 3 in the basic area, were classified as mixed hepatic–renal profiles, consistent with variable contributions from hepatic and renal EPO production.

As a result, we observed a correlation between the progressive shift in EPO profiles and the developmental age, defined as the sum of gestational age at birth and postnatal chronological age (Fig. 1B, Supplementary Table S2). EPO profile distribution differed significantly across developmental age groups (chi-square test of independence, p < 0.0001). The premature newborn group (≤37 weeks of developmental age) was predominantly associated with the foetal profile, whereas older newborns (>49 weeks of developmental age) showed a marked enrichment in the adult profiles.

**Fig. 2:**
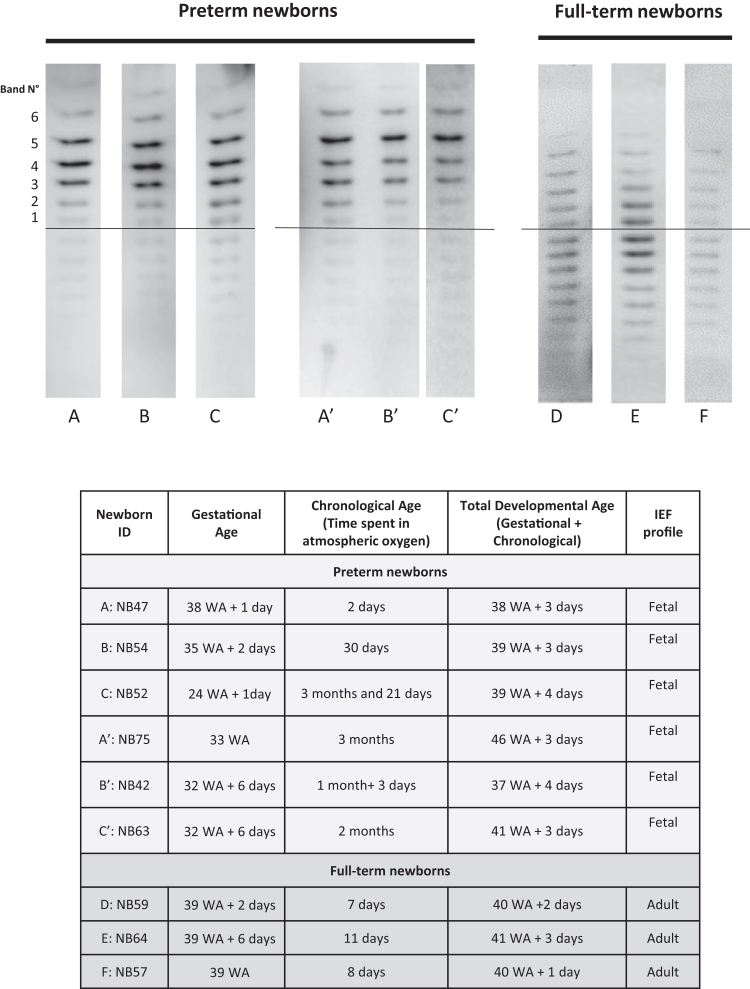
**Analysis of EPO isoforms from newborns with similar developmental age but different durations of postnatal exposure to atmospheric oxygen.** Isoelectric focusing (IEF) electrophoresis showing EPO isoform profiles in preterm neonates with similar total developmental age (A, B, C) or with similar gestational age (A′, B′, C′) and in full-term newborns (D, E, F). The table summarises the age at birth (Gestational age), duration of time spent in atmospheric oxygen (Chronological age) and total developmental age for each newborn.

Interestingly, for newborns with similar total developmental age (subjects A, B and C) or similar gestational age (subjects A′, B′ and C′), the EPO profile did not correlate with the chronological age (the duration of postnatal exposure to atmospheric oxygen); even 2 days to 3 months postnatally, very premature infants retained a foetal-like EPO profile (Fig. 2). Conversely, the full-term newborns presented an adult (renal) profile (subjects D, E and F) (Fig. 2).

### EPO expression dynamics during hiPSC differentiation into liver organoids and relationship to developmental maturation and hypoxia signalling

**Fig. 3:**
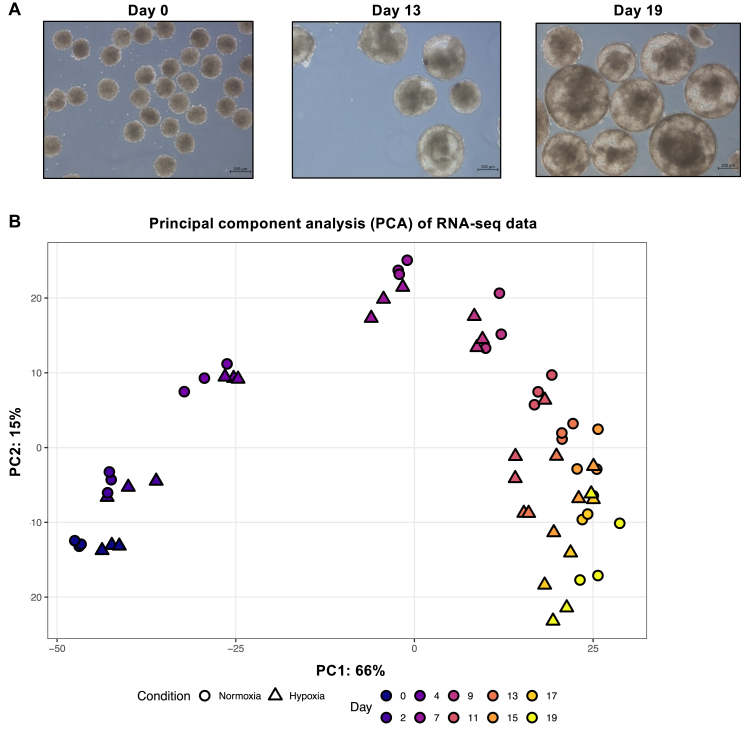

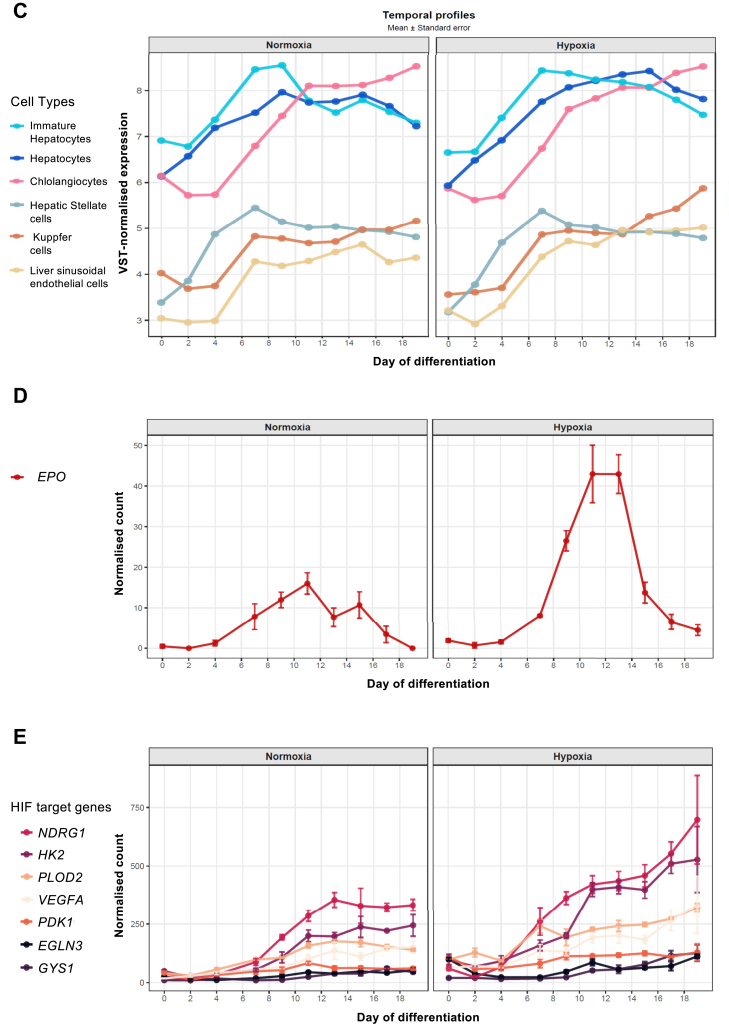
**Quantification of *EPO* expression during the differentiation of hiPSCs into liver organoids.** Data shown represent n = 3 independent organoid differentiations. (A) Representative images of hiPSCs (day 0), immature hepatocytes (day 13), and mature liver organoids (day 19). The scale bar is 200 μm. (B) Principal component analysis (PCA) of 3′ SRP RNA-sequencing. PCA was performed on counts transformed using the Variance Stabilising Transformation (VST) from DESeq2. Each symbol represents one biological replicate (n = 3 per condition and time point). Colours indicate the differentiation time point (days 0, 2, 4, 7, 9, 11, 13, 15, 17 and 19), and shapes denote experimental conditions (circles: normoxia; triangles: hypoxia). The percentage of total variance explained by PC1 and PC2 is indicated on the respective axes. (C) Quantification of markers specific to major hepatic cell populations during organoids differentiation from three independent 3′ SRP RNA-seq experiments. Twenty-four hours prior to collection, organoids were cultured under either normoxic or hypoxic (1% O_2_) conditions. Hepatocyte maturation was initiated on day 7 by switching to Hepatocyte Culture Medium (HCM). Curves represent VST expression to allow comparison on a common scale. Each curve represents the mean expression of marker genes specific to a given cell population. Expression profiles of individual genes are detailed in Supplementary Fig. S1. D) Time-course analysis of *EPO* expression during differentiation from three independent 3′ SRP RNA-seq experiments performed under normoxic or hypoxic conditions. Data are presented as normalised counts. E) Time-course analysis of hypoxia inducible Factor (HIF) target gene expression during differentiation from three independent 3′ SRP RNA-seq experiments. Data are presented as normalised counts. Panel C displays VST expression values, whereas panels D and E show normalised counts (UPM). Different normalisation methods were applied to optimise data visualisation and interpretability for each panel.

To explore the dynamics of *EPO* expression in a simplified *in vitro* model of liver development, we differentiated hiPSCs into three-dimensional liver organoids as described by Harrison et al.,^31^ (Fig. 3A) by performing a time-course analysis. This organoid system has been shown to recapitulate key aspects of hepatic multicellular organisation and progressive maturation, and generates multicellular liver organoids containing multiple hepatic lineage cell types, including hepatocytes, hepatic stellate-like cells, endothelial cells, Kupffer cells, and cholangiocytes. To confirm proper differentiation progression in our experimental conditions, we analysed the average expression of markers representative of major hepatic cell populations throughout differentiation by using 3′-end Sequencing RNA Profiling (3′SRP). PCA of 3′SRP shows that samples primarily segregate by developmental stage, with PC1 (66% variance) reflecting a clear trajectory from early (days 0–4) to more mature stages (days 15–19). Intermediate time points distribute progressively along this axis, indicating continuous maturation. In contrast, normoxia and hypoxia conditions largely overlap within each stage, suggesting that differentiation is the main driver of transcriptomic variation, with limited global impact of oxygen levels. In addition, replicates cluster tightly together, supporting the robustness of the dataset (Fig. 3B). Consistent with progressive hepatic maturation, lineage and functional markers associated with hepatocytes and non-parenchymal hepatic populations showed stable or progressively increasing expression as differentiation progressed (Fig. 3C and Supplementary Fig. S1).

In contrast to these progressive maturation-associated patterns, through transcriptomic profiling across differentiation time points*, EPO* expression displayed a distinct developmental trajectory. We previously showed that immature hepatocytes differentiated from hiPSCs are capable of significantly expressing *EPO*^26^ (two-dimensional culture model of hepatocyte-like cells^35^, ^36^, ^37^). Here, we observed a strong expression of *EPO* during the early stages of hepatocyte differentiation, with a peak around days 11–13, and subsequently declined sharply, reaching minimal levels by late differentiation (day 19), under hypoxic and normoxic conditions (Fig. 3D). Importantly, this EPO trajectory did not correlate with the disappearance or reduction of any specific cellular population within the organoids. Temporal expression profiles of markers associated with the different cellular populations did not mirror the transient *EPO* peak followed by decline. Notably, hepatocyte and hepatic stellate-like cell populations remained detectable at late differentiation stages despite the marked reduction in *EPO* expression. These findings indicate that the decline of *EPO* expression during maturation is not explained by changes in organoid cellular composition but instead reflects intrinsic developmental regulation associated with hepatic maturation.

These results were confirmed by targeted analysis, which demonstrated a statistically significant induction of *EPO* under hypoxia in hepatocytes at days 13 and 15 (Supplementary Fig. S4B obtained from three additional independent differentiation experiments). Notably, as the liver organoids progressed through maturation, *EPO* expression markedly decreased, and by the end of differentiation (days 19–21), they no longer responded to hypoxia with *EPO* induction (Supplementary 2B). This observation was further confirmed using a second hepatocyte maturation protocol (data not shown).^38^

To evaluate whether oxygen sensing remained functional at late differentiation stages, we analysed canonical hypoxia-responsive genes at day 19. Multiple established hypoxia target genes, including *VEGFA, EGLN3, NDRG1, PDK1, HK2,* and others remained inducible under hypoxic condition compared to normoxic condition (Fig. 3E and Supplementary Fig. S3). These results demonstrate that the absence of hypoxia-induced *EPO* expression at late maturation stages reflects selective loss of EPO responsiveness rather than global impairment of hypoxia signalling pathways.

Finally, we examined the temporal expression of transcription factors previously implicated in hepatic *EPO* regulation,^2^ including *GATA4*, *GATA2, HNF4A, FOXA2* and others. None of these transcription factors exhibited temporal dynamics matching the transient peak followed by decline observed for *EPO* expression (Supplementary Fig. S5). In particular, no candidate regulator showed progressive downregulation in parallel with *EPO* extinction during late differentiation. These results suggest that the loss of *EPO* expression during hepatic maturation is unlikely to be explained by changes in the expression of individual transcription factors and instead may reflect broader developmental regulatory remodelling.

To this end, these findings support the hypothesis that the decrease in hepatic *EPO* expression is primarily driven by intrinsic developmental maturation of liver cells rather than by atmospheric oxygen levels, consistent with the existence of a transient developmental window of hepatic *EPO* regulatory competence.

## Discussion

The recent discovery that foetal/hepatic-like EPO exhibits higher bioactivity than adult/kidney-derived EPO^26^ has provided clues for earlier observations in which measured foetal EPO concentrations appeared disproportionately low relative to the high erythropoietic demands during development.^39^ This unique property of foetal EPO, which remains detectable at birth, complements other enhanced erythropoietic features in newborns, such as the increased sensitivity of neonatal erythroid precursors to EPO compared to their adult counterparts^40^ and the high oxygen affinity of HbF.^41^ Given that these properties are closely linked to oxygen and that the regulation of hepatic *EPO* expression is strongly influenced by oxygen levels, it was relevant to explore the impact of oxygen variations; specifically the changes occurring at birth; on *EPO* expression, in relation to intrinsic developmental factors.

The rise in oxygen tension following birth, as the newborn transitions from a low-oxygen intrauterine environment to atmospheric oxygen, is thought to contribute to physiological anaemia of infancy. This condition is characterised by a natural and temporary decrease in Hb and red blood cell levels that typically occurs within the first weeks to months of life. In addition to the anticipated role of increased liver oxygenation, resulting from circulatory adjustments shortly after birth, several other factors contribute to this process. These include the gradual replacement of high-affinity foetal haemoglobin (HbF) with adult haemoglobin (HbA), the shorter lifespan of foetal red blood cells,^24^ and the presumed delay in renal erythropoietin (EPO) production in newborns.^39^

To address this question, we analysed EPO profiles, whose hepatic or renal origin can be distinguished,^26^, ^27^, ^28^^,^^42^^,^^43^ in newborns with varying developmental ages, ranging from 29 to >49 weeks and varying durations of exposure to atmospheric oxygen (from 1 day to 11 months). We demonstrated that the basic EPO IEF profile, classified as “foetal” and corresponding to the hepatic-like EPO, is mostly present in early preterm infants. In contrast, “adult” profiles, indicative of renal origin, were correlated to the developmental age and became predominant after 49 weeks.

In hiPSC-derived mature hepatocytes as a cellular model, kinetic measurement of *EPO* expression and transcriptomic data revealed a dramatic decline in *EPO* expression as maturation progressed, even under hypoxic conditions. Importantly, our data suggest that hepatic *EPO* expression is not solely determined by oxygen sensing capacity but is instead constrained by developmental regulatory state. While canonical hypoxia-responsive pathways remained functional in mature organoids, *EPO* expression became selectively uncoupled from hypoxic stimulation. This observation supports the concept that oxygen-dependent transcriptional activation of the *EPO* locus requires a permissive developmental regulatory environment, likely involving chromatin accessibility and enhancer activity, which is progressively lost during hepatocyte maturation.^44^ Consistent with this interpretation, transcription factors previously implicated in hepatic *EPO* regulation did not exhibit temporal expression patterns paralleling *EPO* decline, suggesting that *EPO* silencing during maturation is unlikely to be explained by changes in individual transcription factor abundance. These findings indicate that *EPO* silencing during hepatic maturation reflects higher-order developmental regulatory mechanisms. Accordingly, the liver organoid model may serve as a valuable system to investigate the factors and mechanisms responsible for the silencing of EPO expression during hepatic maturation.

The present work benefits from a cohort of 89 newborns spanning a wide range of developmental ages and durations of postnatal oxygen exposure, providing a clinically representative dataset to characterize the hepatic-to-renal EPO switch *in vivo*. The combination of a descriptive approach — based on EPO isoelectric focusing profiles — with a mechanistic cellular model offers complementary levels of evidence that reinforce the overall interpretation. In addition, the use of isoelectric focusing to resolve EPO glycoforms provides a functional and tissue-informative readout that extends beyond transcript or protein quantification alone.

A limitation of our study is that longitudinal sampling from the same individual over time was not achievable in practice, as repeated blood sampling after hospital discharge remains logistically and ethically constrained in this case. Hence, the developmental trajectory was approximated from data collected at single time points across distinct newborns. In addition, direct measurement of EPO production at the organ level in living newborns was not feasible for ethical reasons. The hiPSC-derived liver organoid model was therefore used as the closest available surrogate. Although the organoid model used here reproduces key features of hepatocyte maturation, it does not fully capture the complexity of the adult liver, including vascularisation, and systemic endocrine interactions. As such, our findings primarily reflect hepatocyte-intrinsic maturation and regulation and may not fully account for inter-organ crosstalk involved in EPO control *in vivo*.

The developmental *EPO* silencing mechanisms are consistent with broader paradigms of ontogenic gene regulation, in which transcriptional competence is controlled through progressive remodelling of enhancer accessibility and chromatin architecture during tissue development or maturation.^45^ These observations open avenues of research into the molecular mechanisms, particularly the transcriptional and epigenetic mechanisms, responsible for the silencing of *EPO* expression in the liver during development, similar to what has been identified in the regulation of globin genes.^41^ Together, our findings support a model in which hepatic *EPO* expression reflects a transient developmental window of regulatory competence that is progressively restricted during liver maturation, independently of preserved global hypoxia signalling capacity. Identifying these factors could ultimately enable their manipulation to reactivate *EPO* expression in the liver, leading to the generation of endogenous EPO proteins with higher activity.^26^ This would have significant therapeutic implication, notably for the treatment of anaemia associated with renal failure. Moreover, endogenous reactivation of *EPO* expression in the liver may represent a potential alternative to exogenous EPO administration, which has been associated with adverse effects such as hypertension, thromboembolic events, stroke, and increased tumour progression or relapse in certain cancers.^46^, ^47^, ^48^, ^49^

## Contributors

LMa and AM performed IEF experiments and analysed results; AC adapted the protocol for differentiating hiPSC into liver organoids; SI performed experiments on hiPSC, RNA extraction and quantification of *EPO* and hepatic markers expression during the differentiation of hiPSC into liver organoids; EJ performed 3′ RNA-seq data analysis. SI and BG interpreted the 3′ RNA-seq data; FG, NM, DS and LMe provided clinical data and biological samples of neonates. BG, FG, LMa and AM designed the study; BG, FG, LMa, AM, and SI wrote and reviewed the manuscripts. BG, SI, AM, LM and FG have accessed and verified data. All authors contributed to the research and have read and approved the final manuscript.

## Data sharing statement

Raw 3′ RNA-sequencing data generated in this study have been deposited in the European Nucleotide Archive (ENA) under accession number PRJEB111200 (alias: SRP_liver_organoids_8b98822c-f7e2-4a16-80a9-281eb4379d4d). The deposited dataset includes raw FASTQ files.

All other data supporting the findings of this study are available from the corresponding author upon request. Erythropoietin (EPO) isoform profiles and gene expression data from hiPSC-derived hepatocytes can be shared for academic and non-commercial research purposes.

## Declaration of interests

The authors declare no conflicts of interest related to this study.
