# Supplementary material for: Biological maturation drives the hepatic-to-renal switch in erythropoietin production at birth
Source: eBioMedicine. 2026 May 5;127:106277. doi: 10.1016/j.ebiom.2026.106277 (PMC13157183; doi:10.1016/j.ebiom.2026.106277)

Supplemental Data

Supplementary Methods

RNA Sequencing and Read Alignment

Raw sequencing reads were processed using the BiRD bioinformatics pipeline[2]. Reads were demultiplexed and aligned to the human RefSeq reference transcriptome (GRCh38) using BWA. Aligned reads were then parsed to extract and count unique molecular identifiers (UMIs) for each gene in each sample, generating a UMI-corrected gene-level expression matrix for downstream differential expression analysis.

RNA-seq Data Analysis

Transcriptomic data analyses were performed using R software (version 4.3.0) within the RStudio integrated development environment (version 2025.05.1+513). Differential expression analysis was conducted using the DESeq2 package (version 1.50.2)[1], distributed via Bioconductor, complemented by R packages dedicated to data manipulation and visualization, including ggplot2 (version 4.0.1) and viridis for color palettes.

Data Import and Quality Control

Raw count matrices (number of reads per gene per sample) were imported into R and associated with a metadata table describing, for each sample, the sample name, experimental condition (normoxia or hypoxia), the day of differentiation (days 0, 2, 4, 7, 9, 11, 13, 15, 17, and 19), and a corresponding numeric time point index (1 to 10) used to facilitate downstream analyses
Sample were filtered to retain three biological replicates per time point and per condition to ensure a balanced experimental design across all comparisons.
Quality control assessment was performed through exploratory data analysis, including hierarchical clustering and principal component analysis.

Normalization and Modeling with DESeq2

A DESeqDataSet object was constructed using the DESeqDataSetFromMatrix function from the raw count matrix and associated metadata. Two complementary modeling strategies were applied depending on the downstream analysis.

For temporal expression profiling, a simple linear model (design ~ Condition) was specified to account for differences between normoxia and hypoxia conditions. Two normalization approaches were then applied.

First, raw counts were normalized using DESeq2's size factor method to correct for differences in sequencing depth between samples; size factors were estimated using the estimateSizeFactors function, and normalized counts were retrieved using the counts function with the parameter normalized = TRUE. These normalized counts were used for visualizing temporal expression profiles of individual genes and for quantitative inter-sample comparisons. Second, a variance-stabilizing transformation (VST) was applied using the VST function, ensuring that the transformation is performed independently of the experimental design, estimating the variance-mean relationship across all samples without incorporating condition information. VST-transformed data were preferred over raw normalized counts for cell type marker-based population profiling, as they stabilize variance across expression levels and prevent highly expressed genes from masking the temporal dynamics of lowly expressed markers.[1] VST data were therefore used for principal component analysis and for temporal profiles aggregated by cell type.

For pairwise differential expression analysis at specific time points, a full interaction design was implemented using a combined factor representing Condition × Time point (design ~ Condition_Timepoint). This design allowed the use of contrast vectors to perform targeted pairwise comparisons between specific condition–time point groups.

Aggregation by Condition and Time Point

To analyze the kinetics of gene expression over time, summary statistics were calculated for each unique combination of experimental condition and time point for visualization purposes. Specifically, the mean expression across biological replicates, standard deviation (SD), and standard error of the mean (SEM) were computed across the three biological replicates for each condition × time point combination. These statistics were computed for both normalized counts and VST-transformed data, depending on the downstream visualization requirements.

Principal Component Analysis (PCA)

Principal component analysis was performed on VST-transformed data using the plotPCA function from the DESeq2 package. Samples were projected onto the first two principal components (PC1 and PC2), with points colored according to differentiation time point and distinguished by symbol shape according to experimental condition (circles for normoxia,

triangles for hypoxia). The percentage of total variance explained by each principal component was calculated and reported on the corresponding plot axes.

Differential Expression Analysis at Day 19

To compare gene expression between normoxia and hypoxia at the final differentiation time point, a pairwise differential expression analysis was performed at day 19 using the full interaction model (design ~ Condition_Timepoint). The comparison of interest was defined as hypoxia *versus* normoxia at day 19 using a contrast vector. DESeq2 models RNA-seq read counts with a negative binomial generalized linear model and evaluates the statistical significance of model coefficients using Wald tests. Genes were classified according to their adjusted p-value (padj) and log2 fold change (log2FC) into the following categories: significantly upregulated (padj < 0.05 and log2FC ≥ 1), significantly downregulated (padj $<$ 0.05 and log2FC ≤ -1), or non-differential. Mean normalized counts and SEM were calculated across the three biological replicates for each condition and visualized as grouped bar plots with individual data points overlaid.

Selection of Cell Type-Specific Marker Genes

Lists of cell type-specific marker genes were compiled based on published literature and established expression databases. The following gene sets were used to define representative transcriptional signatures of major hepatic cell type populations: hepatocytes (*ALB, HNF4A, TTR, APOA2, TDO2, FOXA2, GLUL, CPS1, PCK1, KRT18, KRT8*), immature hepatocytes (*AFP, PROM1, EPCAM, SOX9*), kupffer cells (*C1QA, C1QB, CD68, CD163, LGALS3, CTSD, CTSB, SPP1*), liver sinusoidal endothelial cells (*LYVE1, PECAM1, KDR, FLT1, CD34*), hepatic stellate cells (*COL1A1, COL3A1, LUM, DCN, PDGFRB, DES, SPARC, COL5A1, COL6A1, THY1, RBP1, VIM, LRAT*), cholangiocytes (*KRT19, KRT7, CFTR, ANXA4, KRT17, SOX9, SLC4A2, SLC4A4*), hypoxia-responsive genes (*NDRG1, HK2, PLOD2, VEGFA, PDK1, EGLN3, GYS1*), erythropoietin (*EPO)*, and transcription factors (*HIF1A, EPAS1/HIF2A, ARNT, BRD4, KCNH2, RXRA, SMAD3, FOXA2, GATA2, GATA4, HNF4A, TBX3*). Average temporal profiles by cell type were obtained by calculating the mean expression of all marker genes within each category at each time point and for each condition. This approach provides a cell type-level signature reflecting the collective behavior of multiple marker genes rather than quantitative estimation of cell abundance or direct cell type deconvolution.

Visualization of Temporal Expression Profiles

All figures were generated using the ggplot2 package in R. Temporal expression profiles are represented as line plots with mean values as data points and error bars indicating SEM. For individual gene profiles, normalized counts or VST-transformed values were plotted against days of differentiation, with one curve per gene and separate facets for each experimental condition. For cell type cluster profiles, the mean VST-transformed expression of all marker genes within each cluster was plotted as a single representative trajectory. Bar plots showing differential expression at day 19 display mean normalized counts ± SEM per condition, with individual replicate values overlaid as data points. Color palettes from the viridis package were used throughout to ensure readability and accessibility.

Legend of Supplementary Tables and Figures:

Supplementary Table 1: Developmental characteristics of the 89 neonatal samples analyzed.

Each sample comes from a different newborn. Gestational age: age at birth calculated in weeks (WA) and days (d) of maternal amenorrhea; chronological age: postnatal exposure to atmospheric oxygen at sample collection (days and months); developmental age sum of gestational age and chronological age, representing the total developmental age. EPO profiles were determined by isoelectric focusing (IEF): fetal (predominantly hepatic), adult (predominantly renal), and intermediate (mixed hepatic and renal). Newborns marked with * are shown in Figure 1; those marked with # in Figure 2. NB52 presented respiratory distress syndrome at birth, progressing to moderate bronchopulmonary dysplasia, with refractory hypoxemia between days 11–15 and EPO treatment from days 14–26 (250 IU/kg, thrice weekly).

Supplementary Table 2: Classification of EPO isoform profiles according to developmental age.

Distribution of the 89 neonatal samples (see Supplementary Table 1) across the three EPO profile categories : fetal (predominantly of hepatic origin); intermediate (mixed hepatic and renal); adult (predominantly renal). Samples are stratified by the developmental age (sum of gestational age and chronological age in weeks and days of maternal amenorrhea) at the time of collection. Percentages and statistics analyses shown in Figure 1B were calculated from these data.

Supplementary Figure 1: Temporal expression profiles of different cell type marker genes during liver organoid differentiation.

The presented results originate from 3′SRP RNA-sequencing transcriptome analysis. For each cell type, the mean expression of its marker genes was calculated at each time point and plotted as a single curve under normoxia (left) and hypoxia (right). Data represent mean ± SEM, n = 3 biological replicates per time point. Expression values are VST (Variance-Stabilizing Transformation) counts, which stabilize variance across expression levels and reduce the dominance of highly expressed genes in downstream visualization.[1] A) Immature hepatocytes. B) Hepatocytes. C) Kupffer cells. D) Liver sinusoidal endothelial cells. E) Hepatic stellate cells. F) Chlolangiocytes.

Supplementary Figure 2: RT-qPCR validation of hepatocyte maturation markers and EPO expression during organoid differentiation.

The presented results originate from independent triplicates, distinct from those used for 3′SRP RNA sequencing. A) Quantification of hepatocyte maturation markers during differentiation under normoxic conditions using TaqMan probes. Hepatocyte maturation was initiated on day 7 by switching to Hepatocyte Culture Medium (HCM). *ALB*, *CYP3A4 and CYP3A7* expression levels were normalized to the average expression of three housekeeping genes using the $2^{-\Delta Ct}$ method. Data are presented as mean ± SEM. Statistical analysis was performed using a repeated-measures one-way ANOVA test. Corresponding p-values are indicated above the data points. B) Time-course analysis of *EPO* mRNA expression during differentiation in three independent experiments (independent triplicates, distinct from those used for 3′SRP RNA sequencing). Twenty-four hours prior to sample collection, organoids were cultured under either normoxic or hypoxic (1% O_2) conditions. *EPO* expression using TaqMan probe was normalized to the average expression of three housekeeping genes using the $2^{-\Delta Ct}$ method.

The upper graph shows individual values obtained over time, with each point representing an independent differentiation experiment. The lower graph presents the statistical analysis of these data. Data are shown as mean ± SEM. Statistical analysis was performed using a two-way ANOVA test. P-values are indicated in red when comparing hypoxic conditions across different time points, and in black when comparing normoxic *versus* hypoxic conditions at a given time point.

Supplementary Figure 3: Differential expression of hypoxia-responsive genes between normoxia and hypoxia at day 19 of liver organoid differentiation.

The presented results originate from 3′SRP RNA-sequencing transcriptome analysis. Bar plots show mean normalized counts ± SEM for each gene under normoxia (blue) and hypoxia (red). Individual data points from the three biological replicates are overlaid on each bar. Statistical significance of differential expression between conditions was assessed using DESeq2 pairwise contrast (hypoxia at day 19 *vs* normoxia at day 19). Level of statistical significance based on adjusted p-value (padj): * padj < 0.05, ** padj < 0.01, *** padj < 0.001, **** padj < 0.0001, ***** padj < 0.00001, ns = not significant. Statistical significance was determined using DESeq2 Wald test (padj).

Supplementary Figure 4: Temporal expression profiles of transcription factors and erythropoietin during liver organoid differentiation under normoxia and hypoxia.

The presented results originate from 3′SRP RNA-sequencing transcriptome analysis. Mean VST (Variance Stabilizing Transformation) expression values ± SEM are shown across 10 differentiation time points (day 0 to day 19; n = 3 independent biological replicates per time point). Each line represents one gene. Left and right panels correspond to normoxia and hypoxia conditions, respectively. A) Transcriptions factors. B) Erythropoietin.

References

1. Love MI, Huber W, Anders S. Moderated estimation of fold change and dispersion for RNA-seq data with DESeq2. *Genome Biol.* 2014;15(12):550.
2. Charpentier E, Cornec M, Dumont S, et al. 3’ RNA sequencing for robust and low-cost gene expression profiling. Published online 28 January 2021. Accessed 10 February 2026. https://www.protocols.io/view/3-rna-sequencing-for-robust-and-low-cost-gene-expr-d3vt8n6n

Supplementary Table 1

**New born tested**	**Gestational Age** (in weeks of amenorrhea (WA) and days (d))	**Chronological Age**	**Age of development at the date of sample collection** (in weeks of amenorrhea (WA) and days (d))	**EPO type according to IEF analysis**
NB1	27 WA + 6d	8 days	**29 WA**	FETAL
NB2	26 WA + 6d	16 days	**29 WA + 1d**	FETAL
NB3	30 WA + 3d	5 days	**31 WA + 1d**	FETAL
NB4	30 WA + 2d	9 days	**31 WA + 4d**	FETAL
NB5	32 WA + 3d	1 day	**32 WA + 4d**	ADULT
**NB6***	32 WA + 1d	4 days	**32 WA + 5d**	FETAL
NB7	32 WA + 5d	1 day	**32 WA + 6d**	FETAL
NB8	33 WA+1d	1 day	**33 WA + 2d**	FETAL
NB9	33 WA + 4d	1 day	**33 WA + 4d**	FETAL
NB10	33 WA + 3d	4 days	**33 WA + 6d**	FETAL
NB11	34 WA	3 days	**34 WA + 2d**	FETAL
NB12	33 WA + 3d	8 days	**34 WA + 4d**	FETAL
NB13	34 WA + 5d	1 day (15H)	**34 WA + 5d**	FETAL
NB14	32 WA + 1d	18 days	**34 WA + 5d**	FETAL
NB15	33 WA + 2d	10 days	**34 WA + 5d**	FETAL
NB16	34 WA + 4d	1 day	**34 WA + 5d**	FETAL
NB17	34 WA + 5d	2 days	**35 WA**	FETAL
NB18	34 WA + 3d	6 days	**35 WA**	FETAL
NB19	33 WA + 4d	11 days	**35 WA + 1d**	FETAL
NB20	33 WA + 6d	9 days	**35 WA + 1d**	FETAL
**NB21***	35 WA + 1d	1 day	**35 WA + 2d**	FETAL
NB22	32 WA + 6d	18 days	**35 WA + 3d**	FETAL
NB23	33 WA + 2d	15 days	**35 WA + 3d**	FETAL
NB24	33 WA + 2d	16 days	**35 WA + 4d**	FETAL
NB25	34 WA + 6d	6 days	**35 WA + 5d**	FETAL
NB26	34 WA + 4d	8 days	**35 WA + 5d**	FETAL
NB27	36 WA + 2d	1 day	**36 WA + 3d**	FETAL
NB28	36 WA + 2d	1 day	**36 WA + 3d**	FETAL
NB29	35 WA +6d	4 days	**36 WA + 3d**	FETAL
NB30	33 WA + 1d	24 days	**36 WA + 4d**	FETAL
NB31	35 WA + 2d	9 days	**36 WA + 4d**	FETAL
NB32	34 WA + 4d	17 days	**37 WA**	FETAL
NB33	34 WA + 4d	17 days	**37 WA**	FETAL
NB34	34 WA + 4d	17 days	**37 WA**	FETAL
NB35	36 WA + 4d	4 days	**37 WA + 1d**	FETAL
NB36	35 WA + 6d	9 days	**37 WA + 1d**	FETAL
NB37	32 WA + 6d	1 month	**37 WA + 1d**	FETAL
**NB38***	33 WA + 4d	25 days	**37 WA + 2d**	FETAL
NB39	34 WA + 6d	18 days	**37 WA + 3d**	FETAL
NB40	34 WA + 1d	23 days	**37 WA + 3d**	INTERMEDIATE
NB41	37 WA + 2d	2 days	**37 WA + 4d**	ADULT
**NB42#**	32 WA + 6d	1 month + 3 days	**37 WA + 4d**	FETAL
**NB43***	33 WA + 3d	1 month + 1 day	**37 WA + 6d**	FETAL

New born tested	Gestational Age	Chronological Age	Age of development at the date of sample collection	EPO type according to IEF analysis
NB44	33 WA + 4d	1 month	**37 WA + 6d**	FETAL
NB45	37 WA + 6d	1 days	**38 WA**	FETAL
NB46	38 WA + 1d	1 days	**38 WA + 2d**	FETAL
**NB47#**	38 WA + 1d	2 days	**38 WA + 3d**	FETAL
NB48	35 WA + 5d	20 days	**38 WA + 4d**	FETAL
NB49	34 WA	1 month + 3 days	**38 WA + 5d**	FETAL
NB50	34 WA + 4d	1 month	**38 WA + 6d**	INTERMEDIATE
NB51	39 WA + 1d	1day	**39 WA + 2d**	FETAL
**NB52#**	24 WA + 1d	3 months + 21 days	**39 WA + 4d**	FETAL
NB53	39 WA + 3d	1 day	**39 WA + 4d**	FETAL
**NB54#**	35 WA + 2d	1 month	**39 WA + 3d**	FETAL
NB55	35 WA + 3d	1 month	**39 WA + 5d**	FETAL
NB56	35 WA + 3d	1 month	**39 WA + 5d**	INTERMEDIATE
**NB57#**	39 WA	8 days	**40 WA + 1d**	ADULT
NB58	38 WA + 3d	13 days	**40 WA + 2d**	FETAL
**NB59#**	39 WA + 2d	7 days	**40 WA + 2d**	ADULT
NB60	38 WA + 1d	19 days	**40 WA + 6d**	ADULT
**NB61***	39 WA	14 days	**41 WA**	INTERMEDIATE
**NB62***	39 WA + 6d	9 days	**41 WA + 1d**	FETAL
**NB63#**	32 WA + 6d	2 months	**41 WA + 3d**	FETAL
**NB64#**	39 WA + 6d	11 days	**41 WA + 3d**	ADULT
NB65	39 WA + 2d	16 days	**41 WA + 4d**	FETAL
NB66	33 WA	2 months	**41 WA + 4d**	INTERMEDIATE
**NB67***	41 WA + 6d	2 days	**42 WA + 1d**	INTERMEDIATE
NB68	41 WA + 6d	3 days	**42 WA + 2d**	INTERMEDIATE
NB69	42 WA	2 days	**42 WA + 2d**	FETAL
**NB70***	38 WA + 1d	30 days	**42 WA + 3d**	INTERMEDIATE
NB71	40 WA + 6d	12 days	**42 WA + 4d**	FETAL
NB72	39 WA + 3d	28 days	**43 WA + 3d**	FETAL
**NB73***	35 WA + 6d	2 months	**44 WA + 3d**	INTERMEDIATE
NB74	39 WA + 1d	1 month + 9 days	**44 WA + 4d**	INTERMEDIATE
**NB75#**	33 WA	3 months	**46 WA + 3d**	FETAL
NB76	ND (>30 WA)	4 months	**47 WA + 1d**	INTERMEDIATE
NB77	ND (>30 WA)	4 months	**47 WA + 1d**	INTERMEDIATE
NB78	ND (>30 WA)	5 months	**51 WA + 3d**	ADULT
**NB79***	38 WA + 4d	14 weeks	**52 WA + 4d**	ADULT
**NB80***	41 WA	4 months	**58 WA + 3d**	ADULT
NB81	ND (>30 WA)	7 months	**60 WA**	INTERMEDIATE
NB82	41 WA	7 months	**71 WA**	ADULT
**NB83***	37 WA	10 months	**79 WA + 6d**	ADULT
**NB84***	40 WA	10 months	**82 WA + 6d**	ADULT
NB85	40 WA + 6d	11 months	**88 WA**	INTERMEDIATE
NB86	ND	23 days	**23d**	ADULT
NB87	ND	11 months	**330d**	ADULT
NB88	ND	2 months	**60d**	ADULT
NB89	ND	19 weeks	**133 WA**	ADULT

Supplementary Table 2

**Developmental Age**	**Fetal**	**Mixed**	**Adulte**
age ≤ 37 weeks (n=34)	33	0	1
37 weeks < age ≤ 41 weeks (n=27)	19	4	4
41 weeks < age ≤ 49 weeks (n=16)	7	8	1
age > 49 weeks (n=12)	0	2	10

Supplementary Figure 1

A

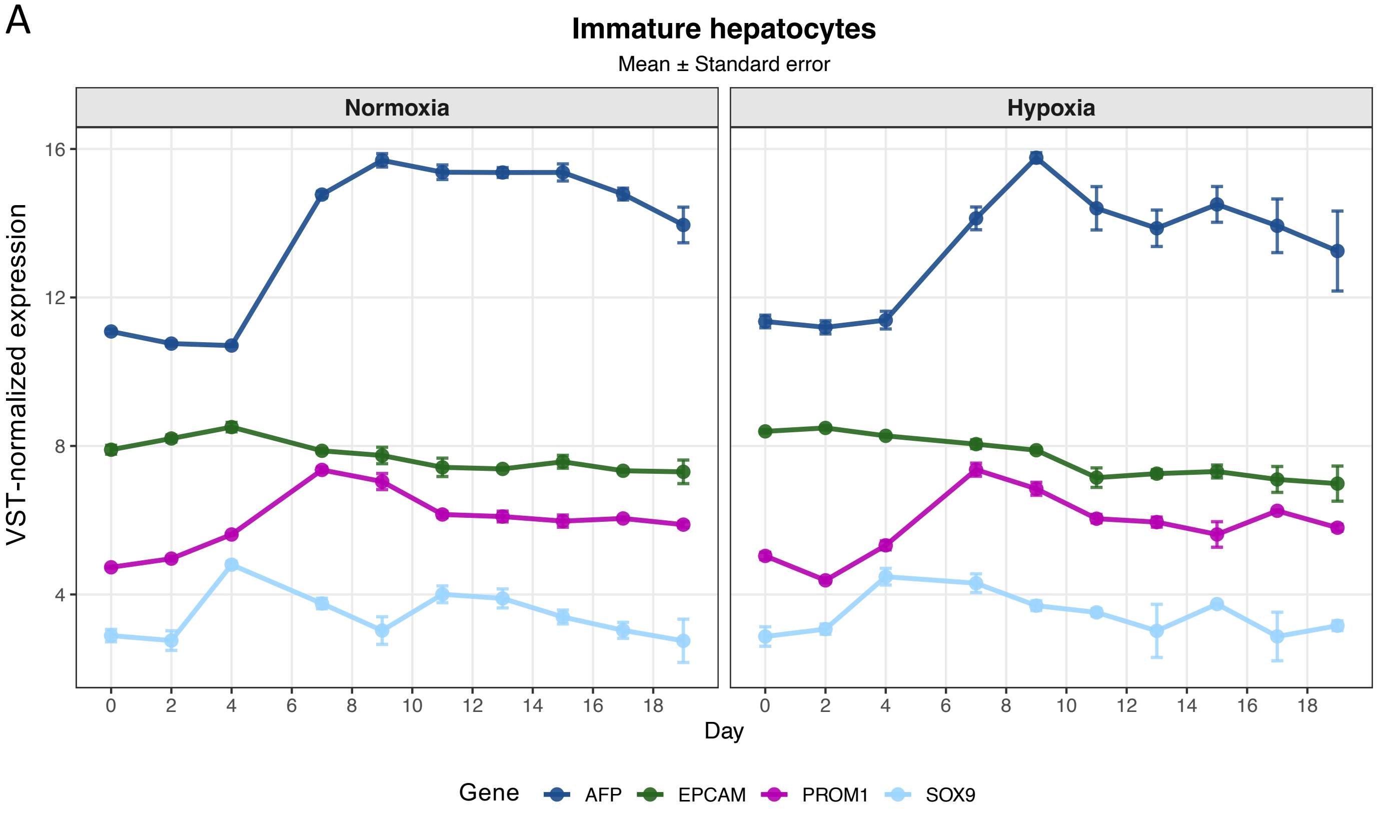

B

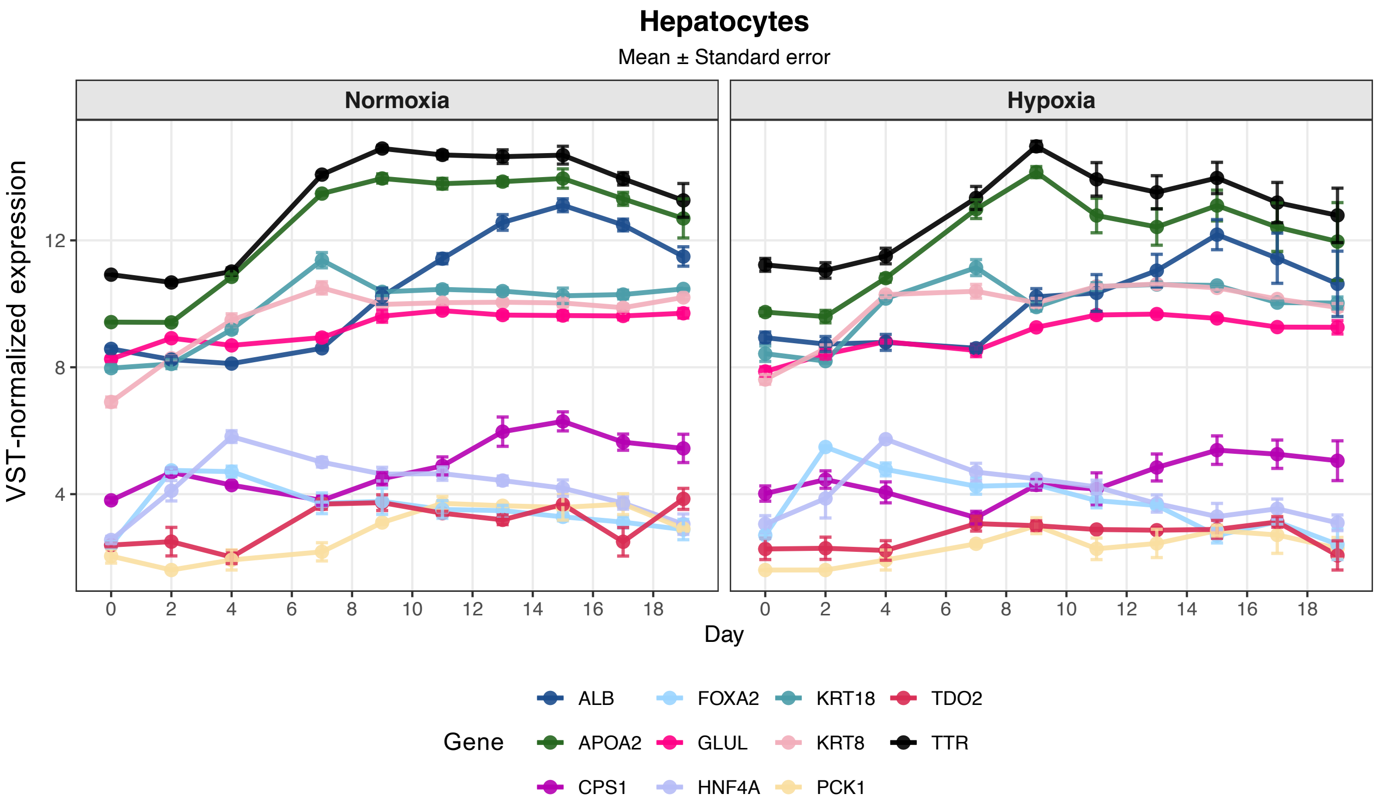

C

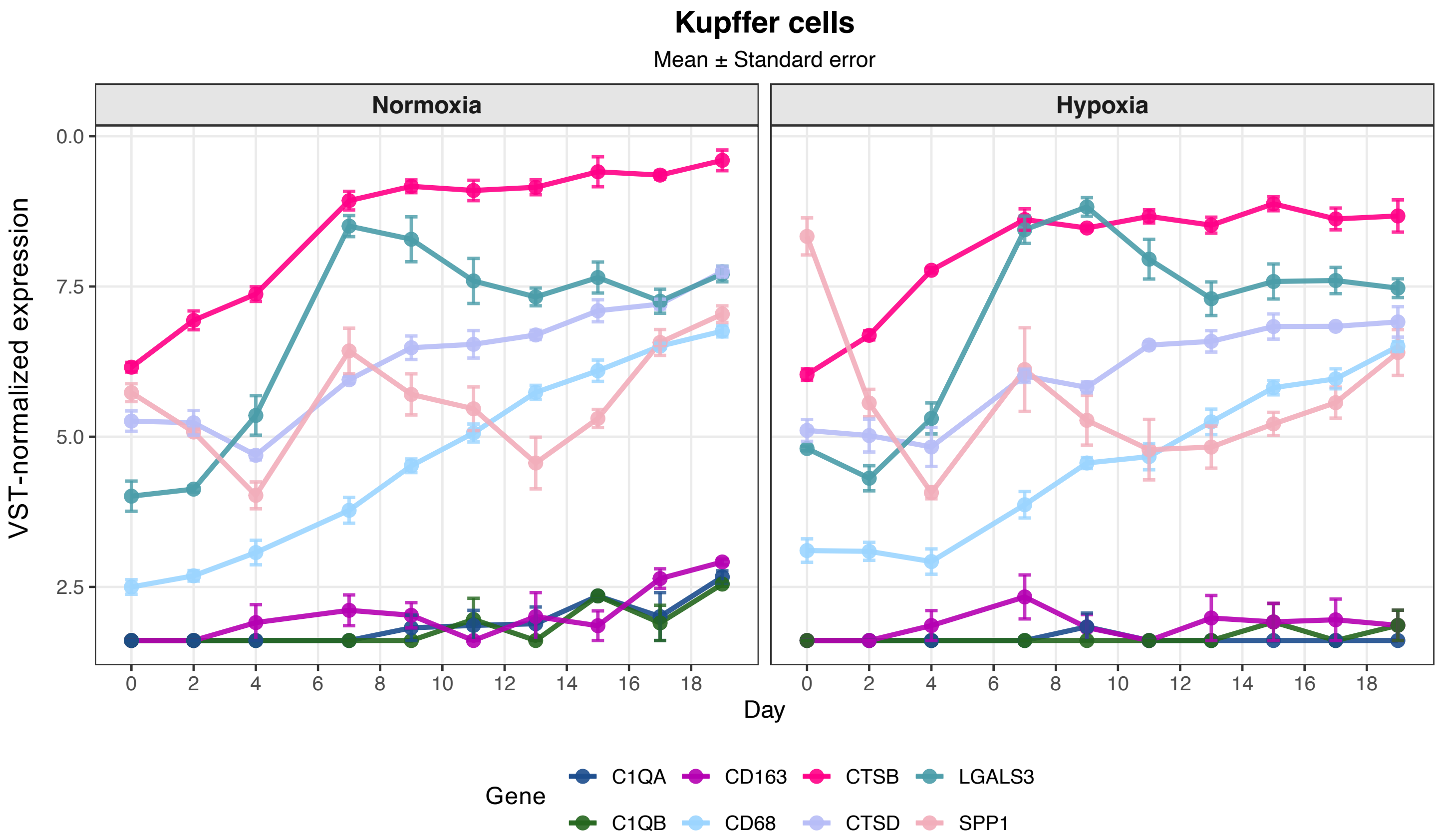

Kupffer cells
Mean ± Standard error
Normoxia
Hypoxia
VST-normalized expression
0.0
7.5
5.0
2.5
0
2
4
6
8
10
12
14
16
18
Day
Gene
C1QA
CD163
CTSB
LGALS3
C1QB
CD68
CTSD
SPP1

D

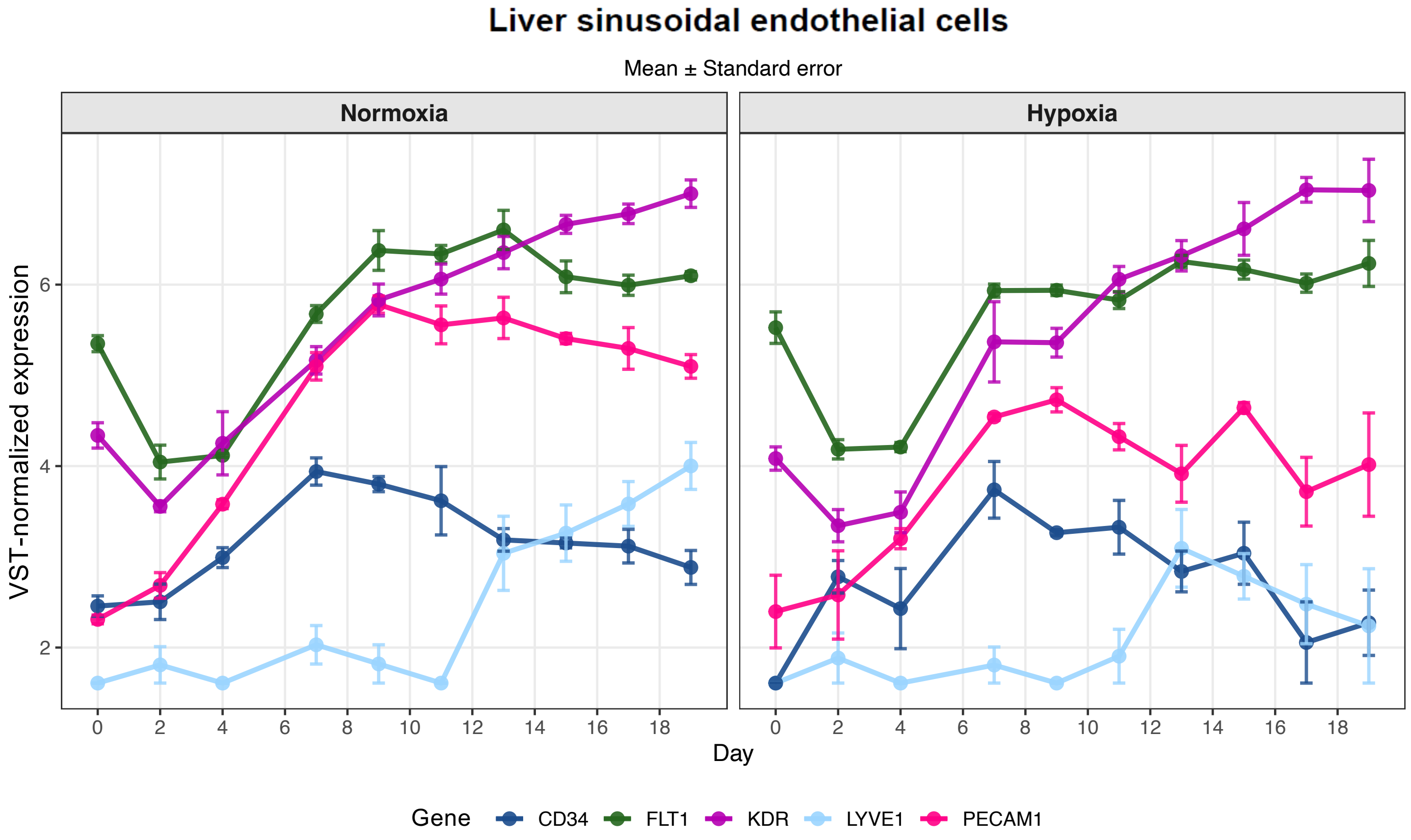

Liver sinusoidal endothelial cells
Mean ± Standard error
Normoxia
Hypoxia
VST-normalized expression
6
4
2
0
2
4
6
8
10
12
14
16
18
Day
Gene
CD34
FLT1
KDR
LYVE1
PECAM1

E

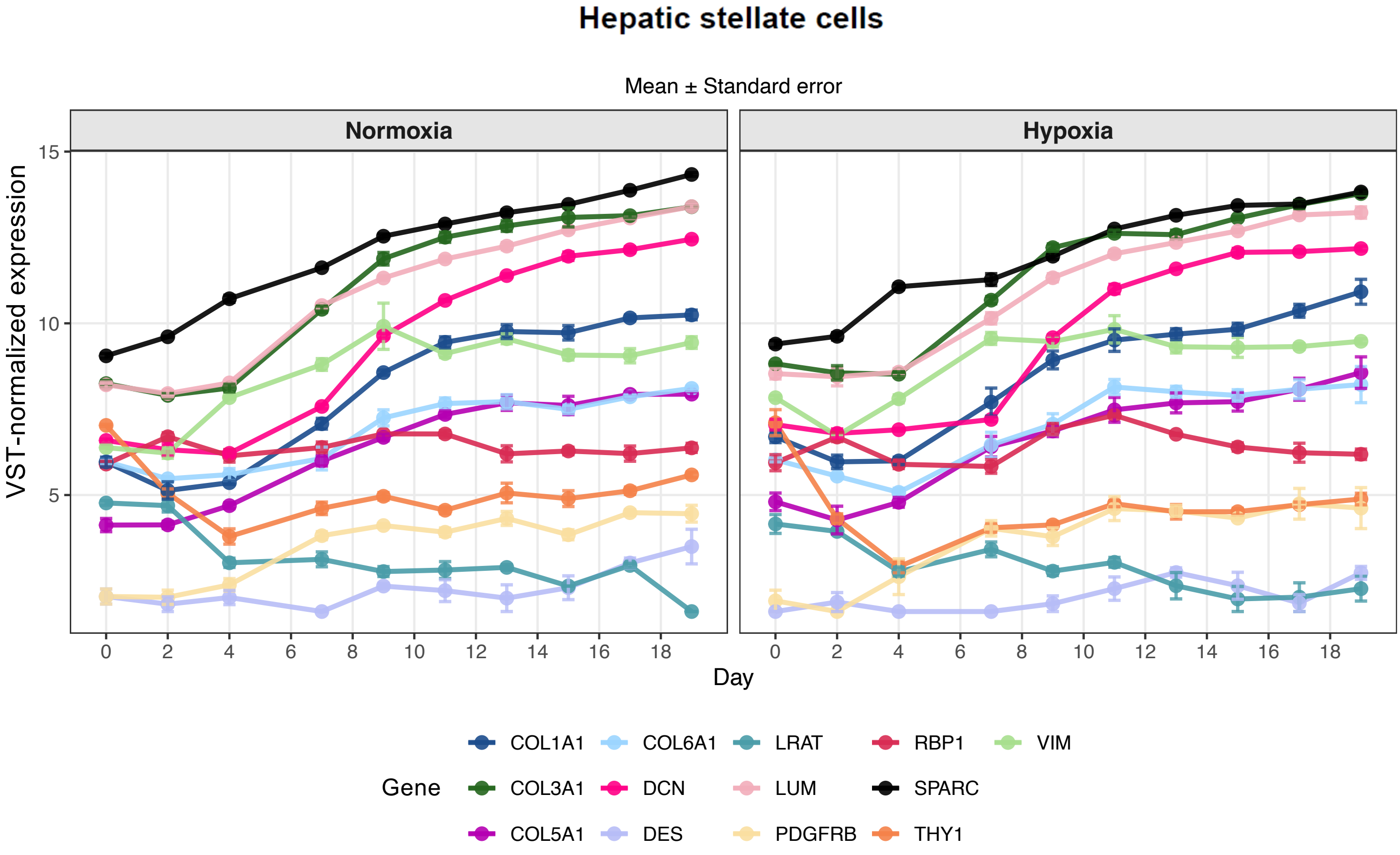
Hepatic stellate cells
Mean ± Standard error
Normoxia
Hypoxia
VST-normalized expression
Day
Gene
COL1A1
COL3A1
COL5A1
COL6A1
DCN
DES
LRAT
LUM
PDGFRB
RBP1
SPARC
THY1
VIM

F

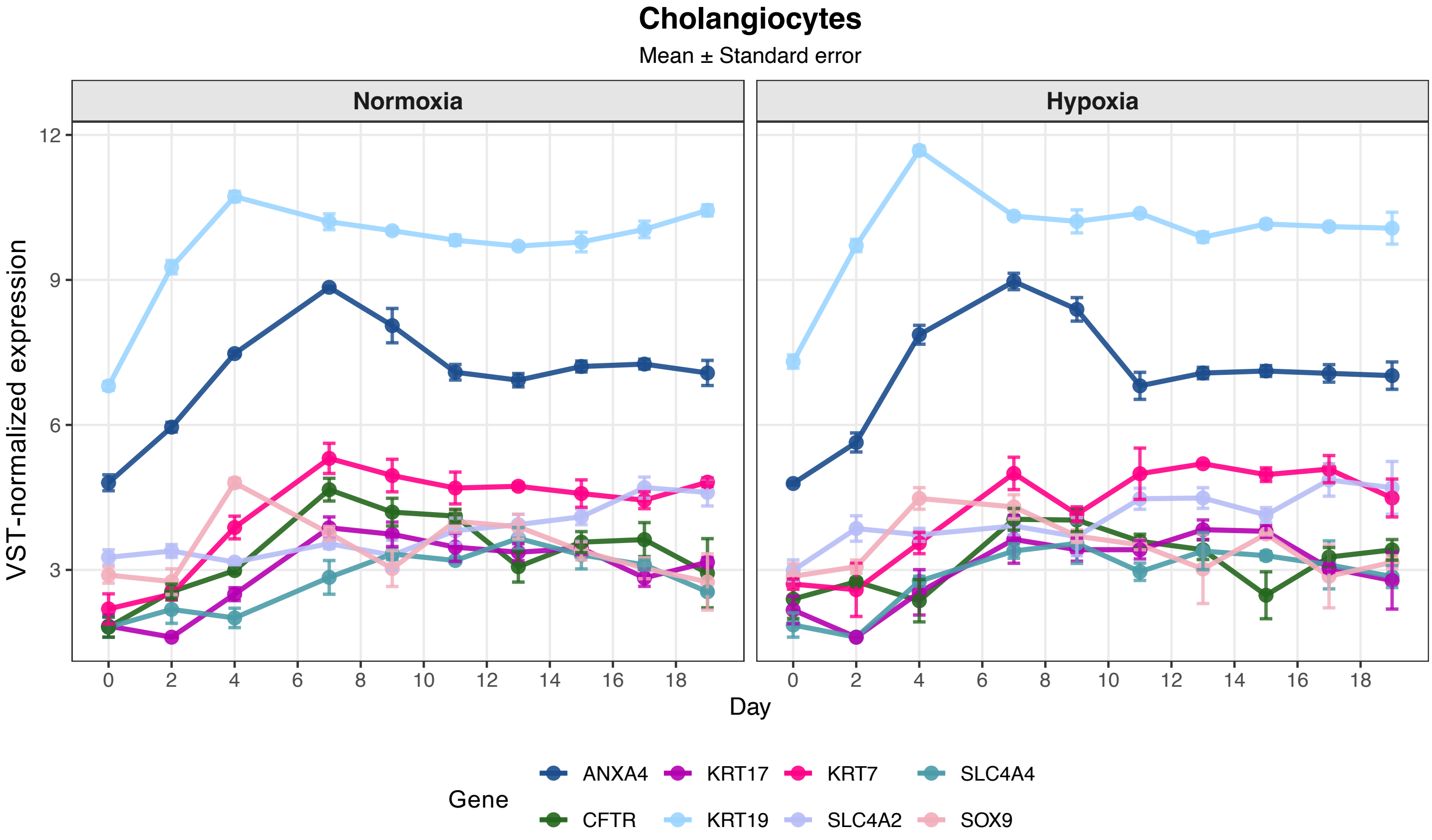
Cholangiocytes
Mean ± Standard error
Normoxia
Hypoxia
VST-normalized expression
Day
Gene
ANXA4
CFTR
KRT17
KRT19
KRT7
SLC4A2
SLC4A4
SOX9

Supplementary Figure 2

A

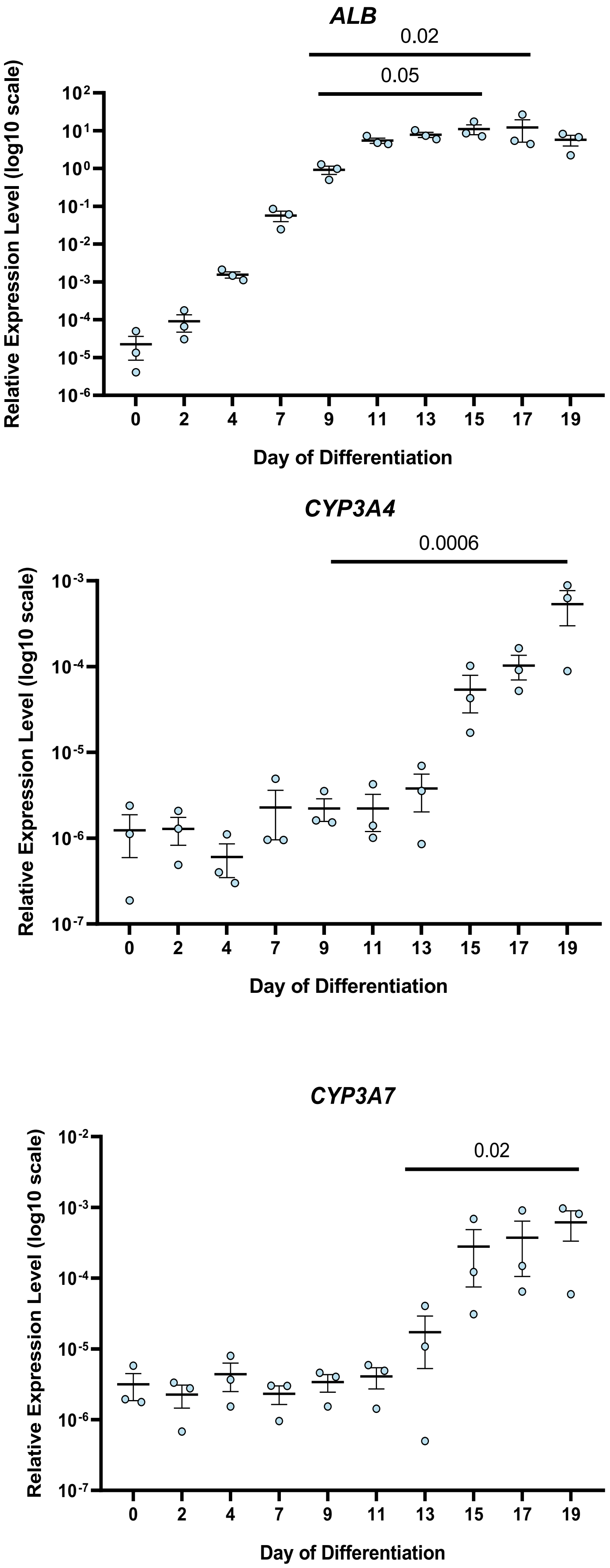

B

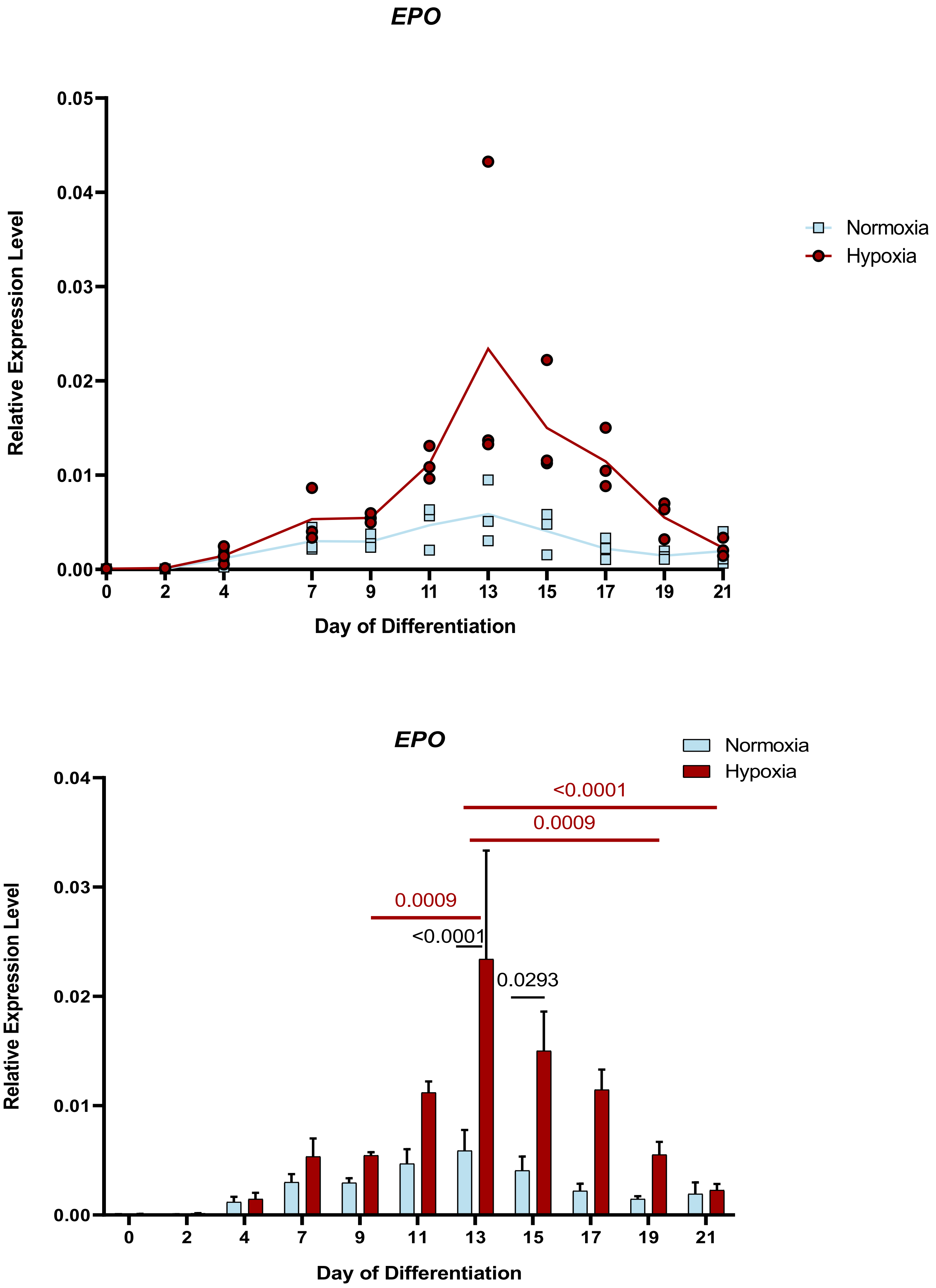
EPO
Relative Expression Level
0.05
0.04
0.03
0.02
0.01
0.00
0
2
4
7
9
11
13
15
17
19
21
Day of Differentiation
Normoxia
Hypoxia
EPO
Normoxia
Hypoxia
<0.0001
0.0009
0.0009
<0.0001
0.0293
Relative Expression Level
0.04
0.03
0.02
0.01
0.00
0
2
4
7
9
11
13
15
17
19
21
Day of Differentiation

Supplementary Figure 3

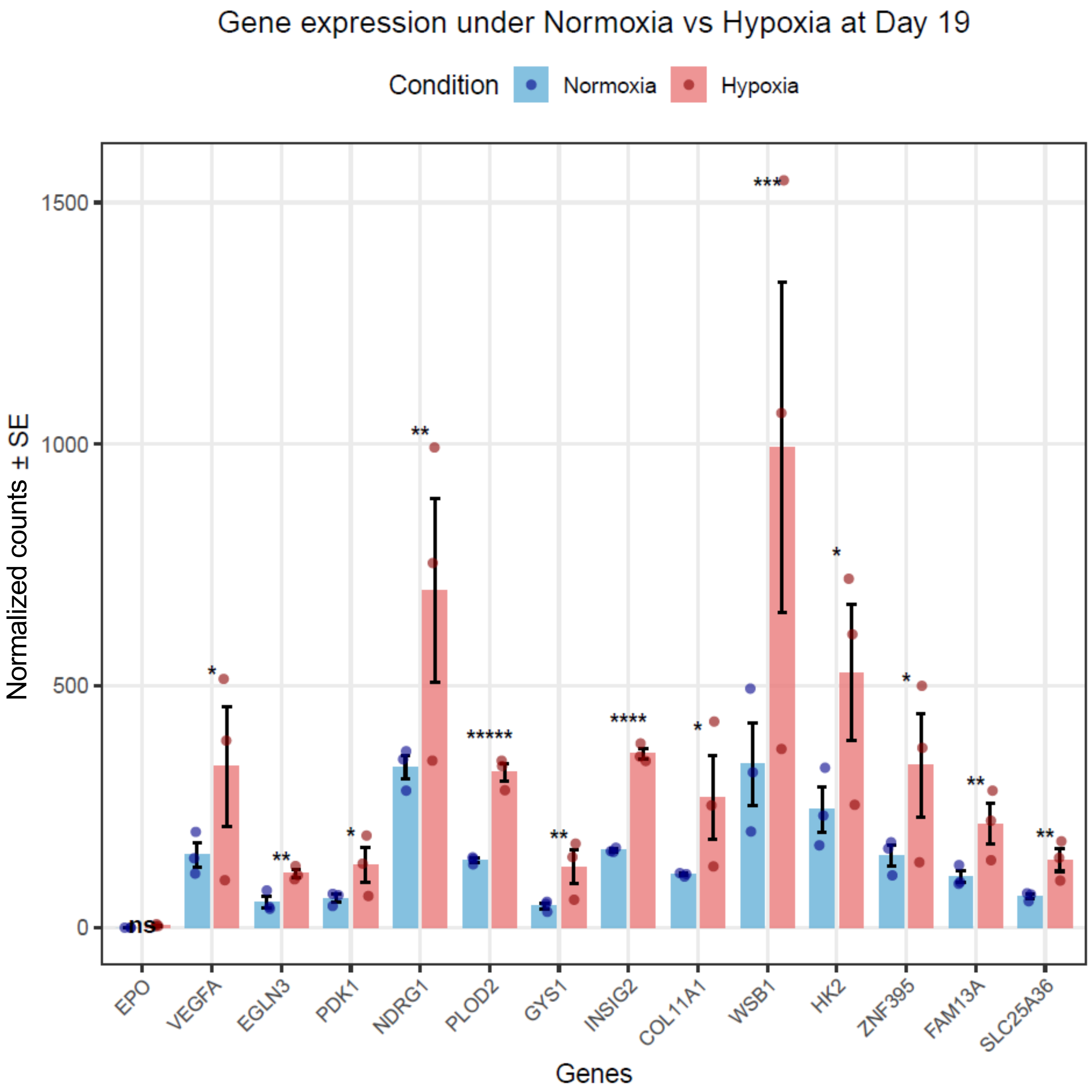

Supplementary Figure 4

A

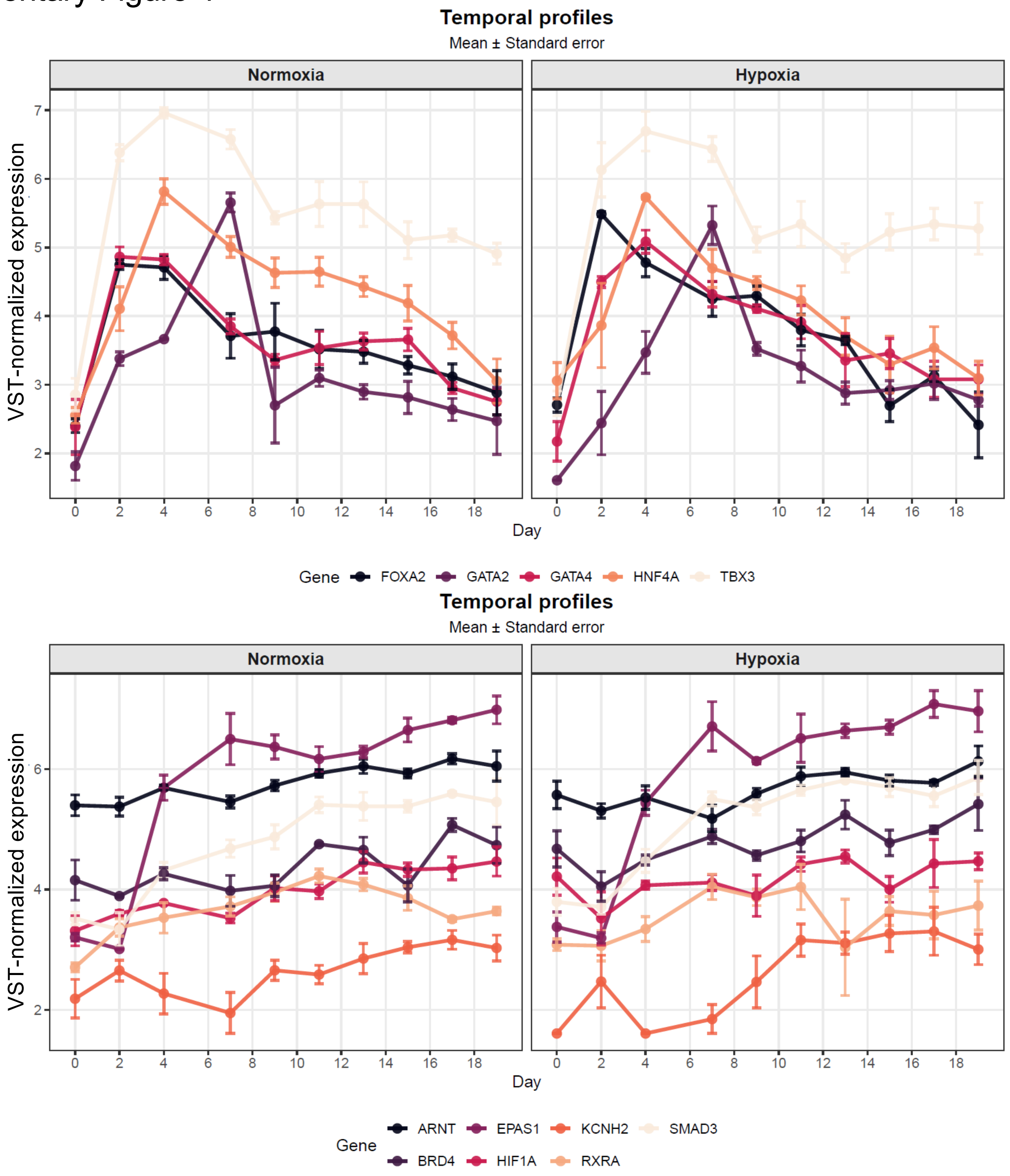

B

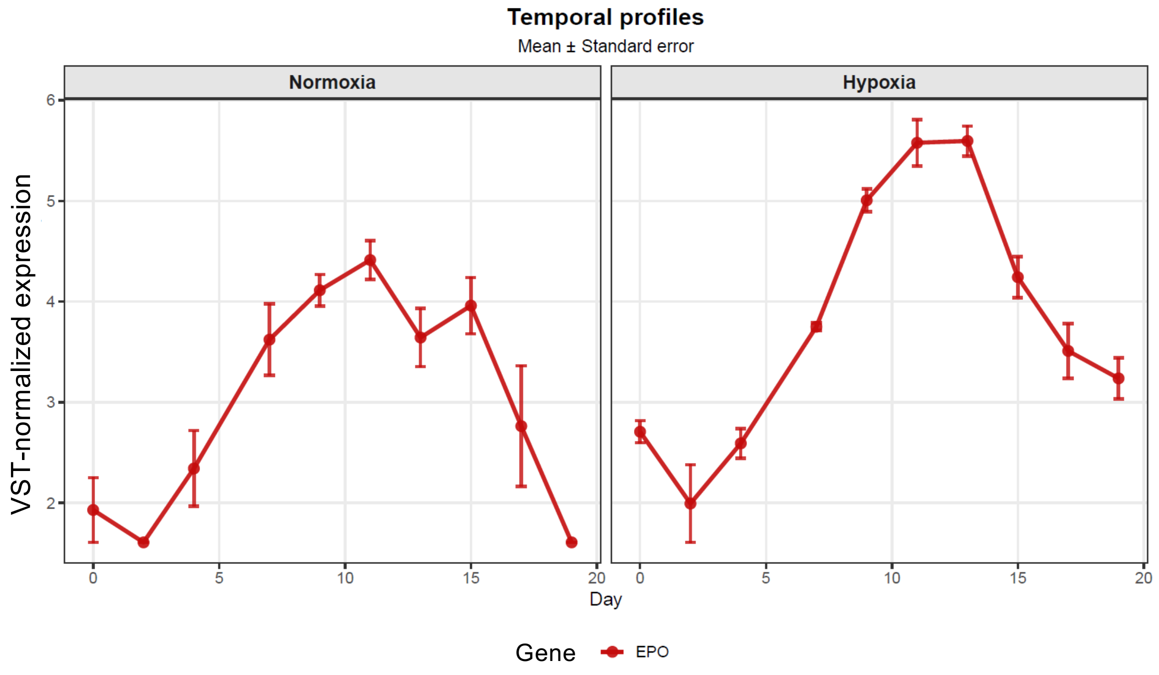

Supplement: Supplementary Data [file mmc1.pdf]
